# Cost-utility of a visiting service for older widowed individuals: Randomised trial

**DOI:** 10.1186/1472-6963-8-128

**Published:** 2008-06-12

**Authors:** Simone Onrust, Filip Smit, Godelief Willemse, Jan van den Bout, Pim Cuijpers

**Affiliations:** 1 Netherlands Institute of Mental Health and Addiction (Trimbos-instituut), P.O. Box 725, 3500 AS, Utrecht, The Netherlands; 2 Department of Clinical Psychology, VU University, Amsterdam, The Netherlands; 3 Department of Clinical and Health Psychology, Utrecht University, Utrecht, The Netherlands

## Abstract

**Background:**

Despite a growing understanding of the effectiveness of bereavement interventions and the groups that benefit most from them, we know little about the cost-effectiveness of bereavement interventions.

**Methods:**

We conducted a cost-utility analysis alongside a randomized clinical trial on a visiting service for older widowed individuals (n = 110) versus care as usual (CAU; n = 106). The visiting service is a selective bereavement intervention that offers social support to lonely widows and widowers by a trained volunteer. Participants were contacted 6–9 months post-loss. Eleven percent of all contacted persons responded and eight percent participated in the trial. The primary outcome measure was quality adjusted life years (QALYs) gained (assessed with the EQ-5D), which is a generic measure of health status. Costs were calculated from a societal perspective excluding costs arising from productivity losses. Using the bootstrap method, we obtained the incremental cost utility ratio (ICUR), projected these on a cost-utility plane and presented as an acceptability curve.

**Results:**

Overall, the experimental group demonstrated slightly better results against slightly higher costs. Whether the visiting service is acceptable depends on the willingness to pay: at a willingness to pay equal to zero per QALY gained, the visiting service has a probability of 31% of being acceptable; beyond €20,000, the visiting service has a probability of 70% of being more acceptable than CAU.

**Conclusion:**

Selective bereavement interventions like the visiting service will not produce large benefits from the health economic point of view, when targeted towards the entire population of all widowed individuals. We recommend that in depth analyses are conducted to identify who benefits most from this kind of interventions, and in what subgroups the incremental cost-utility is best. In the future bereavement interventions are then best directed to these groups.

**Trial registration:**

Controlled trials ISRCTN17508307

## Background

The death of a spouse can lead to serious psychological problems [1], although the impact of spousal bereavement on mental health diverges among the widowed. Targeted support can have a preventive effect and reduce psychological problems resulting from the death of the spouse [2-5]. However, not all studies on bereavement interventions demonstrate positive results [6-8]. Bereavement interventions are defined as all interventions developed to benefit bereaved persons in terms of alleviating the emotional and practical problems following the loss of a loved one. Types of intervention can vary from self-help groups to psychotherapy. It appears that the effectiveness of a bereavement intervention is largely determined by the population towards it is directed. Most outreaching preventive interventions for all widowed individuals are not very beneficial [9], probably because most of the widowed are able to adjust relatively well over time and do not need a specific intervention to regain pre-bereavement levels of functioning. However, this does not apply to all widowed individuals. Several widowed individuals are not able to deal with the loss on their own, and for those widows and widowers a bereavement intervention could be very helpful. Interventions directed towards widows and widowers with a high risk profile, such as having more severe psychological problems or symptoms of complicated grief, do show desirable results [9].

Despite a growing understanding of the effectiveness of bereavement interventions and the groups that benefit most from them, we know little about the cost-effectiveness of bereavement interventions. It is reasonable to assume that some interventions, especially those provided by volunteers, could be cost-effective – even when no superior clinical results could be demonstrated. It is plausible that widows and widowers that have been offered targeted support by volunteers will make less use of health care services, the latter being considerably more expensive than the attention of (trained) volunteers. In order to test this hypothesis, we conducted a cost-utility analysis alongside a randomized clinical trial on a visiting service for older widowed individuals, which failed to demonstrate superior clinical effects on depression of targeted support by trained volunteers over care as usual (Onrust, Willemse, van den Bout & Cuijpers, in press.).

## Methods

### Sample and setting

This cost utility analysis was based on a randomized controlled study on the effects of a visiting service for older widowed individuals. The research has been judged and ethically approved by the METiGG, a medical-ethical committee for research in mental health care settings in the Netherlands. The study was conducted in 18 municipalities in the Netherlands. Making use of the Registry Office, letters were sent to all residents at the age of 55 and older who had lost their spouse 6 – 9 months earlier. This is consistent with the recruitment procedure of most visiting services in the Netherlands. Respondents were contacted only 6 – 9 months after the loss, because in the initial stage of bereavement, social support is usually available in the direct environment of family, friends and neighbours. The letters contained information about the study, an informed consent form, and a short screenings questionnaire. In order to increase study participation, we used local media to inform the population and stimulate application. This media attention resulted in several participants who had not yet received a letter because the death was more recent than 6 months, or who had reconsidered participation several months after they were contacted. This media attention also resulted in 5 participants (2,3%) slightly younger than 55 years of age. Since it is not unconventional that slightly younger individuals make an appeal to the visiting service, and since age did not predict the effects of the visiting service (Onrust & Willemse et al., in press), these respondents were included in the study.

To be eligible for the study, respondents had to meet the following inclusion criteria: widowed during the past year, moderate or strong feelings of loneliness, and the absence of a full-blown mental disorder. In addition, respondents had to be capable of participating in a 1-hour-long interview. Further explanation of the inclusion criteria is presented elsewhere (Onrust & Willemse et al., in press).

**Figure 1:**
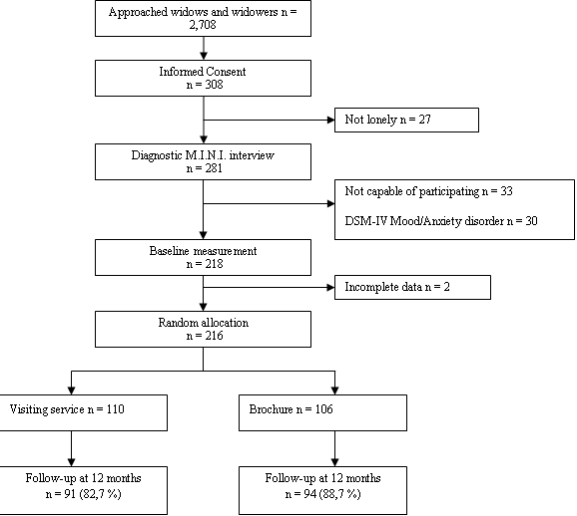
Participants flow through the study.

Figure 1 presents the several steps of the recruitment of study participants. During the one year recruitment period, a total of 2,708 letters were sent to widowed individuals. In total, 308 widowed individuals (11,4%) returned the informed consent form. In order to determine whether the widowed individuals agreeing to participate were eligible for the study, we carried out a stepwise screening procedure.

At first, we measured feelings of loneliness with the 'Loneliness Scale' [10], which was enclosed in the request for participation. Only respondents with at least moderate feelings of loneliness were included in the study. We excluded respondents without feelings of loneliness because the intervention was designed for widowed individuals with social support deficits and because bereavement interventions directed towards the entire population of widowed individuals are generally not effective. Of the 308 widowed individuals agreeing to participate, 27 widows and widowers (8,8%) were excluded because they did not report feelings of loneliness.

All other candidates were contacted by phone for a screening interview, in order to ascertain the capability of engaging in a 1-hour-long interview and the absence of a full-blown mental disorder. The presence or absence of full-blown mental disorders was measured with the M.I.N.I. Plus, a short standardized diagnostic interview [11]. A total of 33 widowed individuals (10,7%) were excluded from the study, as they were considered to be not capable of participating, mostly because they were confused and did not understand the objectives of the study. Based on the M.I.N.I., another 30 widows and widowers (9,7%) were excluded as they appeared to meet the DSM-IV diagnostic criteria for either depression or an anxiety disorder. The remaining 218 widowed individuals (71% of those agreeing to participate; 8,1% of all persons approached) met inclusion criteria and were approached for the baseline measurement. This measurement was completed by 216 widowed individuals who were randomly allocated to the visiting service (n = 110) or a brief brochure (n = 106). The randomization was carried out centrally, using blocked randomization stratified for gender and region with the widowed individual as unit of randomization, with blocks of two widowed individuals. Data were collected at baseline, at 6 months, 12 months and 24 months after baseline. Although all follow-up assessments were attempted to be scheduled as close as possible to the intended point in time, we did allow a deviation of (± 2 weeks). This paper focuses on the cost-utility of the visiting service at the 12 months follow-up assessment, as the intervention was administered during the first 12 months of the study and potential shifts in health care utilization were most likely to occur during this period. At the 12 month follow-up assessment 185 (86%) widows and widowers were retained in the trial. The recruitment of participants and the baseline measurements took place in 2003–2004, data collection for the 12 month follow-up assessment was carried out in 2004–2005.

### Intervention

The experimental intervention was the visiting service, based on the Widow-to-Widow program [12]. Respondents who were allocated to the visiting service were offered 10 – 12 home visits by a trained volunteer. During the home visits, one-to-one support was offered by exchanging experiences and emotions. The volunteers provided the respondents with the opportunity to express their feelings and a better understanding of their grieving process. In addition, the volunteers provided information and sometimes practical help. All volunteers were widowed themselves for some years. They had attended a course of 6 meetings, in which both theoretical knowledge (grief phenomena; tasks of grief; loneliness and social support) and practical skills (empathic listening; conversation techniques; setting boundaries) were learned. Mainly based on the way they participated in this course, their eligibility for the program was evaluated. During the period of home visits, all volunteers were supervised by the coordinator of the visiting service. The coordinators of all visiting services had also attended a course of 6 meetings. In this course, which was based on the "Manual Visiting Services" [13], information was provided on the organization and procedure of the visiting service and the supervision of the volunteers.

Respondents who were allocated to the visiting service were allowed to use all other types of health services and community resources during the intervention period.

The comparison (control) intervention consisted of a brief brochure on depressive symptoms. The brochure provided information and several tips to improve well-being. Respondents who were allocated to the comparison group were not offered any type of intervention, but were allowed to use all types of health services and community resources with the exception of the visiting service during the study. Mostly, widowed individuals are supported by their direct environment. This support generally diminishes over time. Although there are several interventions available for widowed individuals to cope with their grief, it depends on the widowed individual whether he or she will actually use the available services. Generally, only a small amount of the widowed population does make use of special services.

### Clinical end terms

Quality of life was assessed with the EuroQol (EQ-5D) [14]. The EuroQol is made up of five dimensions: Mobility, Self-care, Usual Activities, Pain/Discomfort, and Anxiety/Depression. Respondents were asked to indicate for each dimension whether they experienced 'no problems', 'some problems', or 'extreme problems'. Subsequently, the separate scores were combined into the EQ-5D Index, a health status index. The EQ-5D Index can be linked directly to empirical values for health status of the general public, which allows the conversion to utilities [15].

### Resource use

For this study we adopted a societal perspective, including the cost of all types of health care services (direct medical costs), patient costs such as costs for traveling and parking (direct non-medical costs) and costs deriving from not being able to perform domestic tasks. We did not include costs attributable to productivity loss, since our sample consisted of older widowed individuals of which only a small part (14%) was employed at baseline. The number of widowed individuals that did report absence from work or reduced efficiency at work (3% at baseline and 1% at follow-up) was too small to be taken into account. Information on the use of health care services and the capability of performing domestic tasks was gathered with parts of the Trimbos and institute of Medical Technology Assessment Questionnaire on Costs Associated with Psychiatric Illness (TiC-P) [16].

Direct medical and direct non-medical costs are presented in Table 1. Direct medical costs are treatment costs for several formal (e.g. general practitioners, mental health services, social work, home care) and informal caregivers (such as family and friends), which were calculated by multiplying the number of health service units (e.g. consultations, contacts) by their standard cost price [17]. Since the TiC-P measures health care utilization during the past 4 weeks, the costs were subsequently converted to annual costs. We also included the costs of antidepressant, anxiolytic and hypnotic medication, calculated as the price per standard daily dose as reported in the Pharmaceutical Compass [18], multiplied by the number of prescription days, plus the pharmacist's dispensing costs of €6,45 per prescription. Since most psychiatric drugs are prescribed for a period of three month on average, we added the pharmacist's dispensing costs 4 times in order to estimate annual costs. Medication use was assessed by the interviewer, who asked the respondents what kind of prescription drugs they used. Participants were encouraged to get the box of the medication in order to ascertain the correct name of the drug. At last, costs arising from being too ill to perform domestic tasks were evaluated at the price of domestic help at €8,30.

**Table 1:** Direct medical and direct non-medical costs by health service type

	**Direct Medical Costs (in 2003 €)**		**Direct Non-Medical Costs (in 2003 €)**	
**Health service type**	**unit**	**cost price^a^**	**km, P, hrs^b^**	**cost price^c^**

Medical doctor	Consult	20,20	1.8 km, 1 h	11,10
Medical specialist	Consult	98,00	7 km, 2 h	20,20
Regional mental health service	Contact	124,00	10 km, 3 h	29,00
Regional addiction service^d^	Contact	124,00	10 km, 3 h	29,00
				
Mental Hospital – Outpatient	Consult	88,00	12 km, 4 h	37,20
Mental Hospital – Day care	Contact	125,00	12 km, 4 h	37,20
Mental Hospital – Inpatient	Day	250,00	8 h	66,40
				
General Hospital – Outpatient	Consult	56,00	7 km, 3 h	28,50
General Hospital – Day care	Contact	229,00	7 km, 4 h	36.80
				
Teaching Hospital – Outpatient	Consult	100,00	12 km, 3 h	29,30
Academic Hospital – Day care	Contact	229,00	12 km, 4 h	37,60
				
Private practice psychotherapist	Session	76,00	5 km, 2 h	19,90
Social worker^e^	Contact	45,00	7 km, 3 h	28,50
Physiotherapist	Contact	22,75	1,8 km, 2 h	19,40
Alternative Healer	Contact	8,30	1,8 km, 2 h	19,40
Self-Help	Session	0,00	10 km, 3 h	29,00
				
Home care, nursing	Hour	30,70	0 km, 0 h	0,00
Home care, domestic	Hour	21,70	0 km, 0 h	0,00
Informal care (family, friends)^f^	Hour	8,30	0 km, 0 h	0,00

^a ^Integral unit cost prices [17].

^b ^Based on average distances (in km), parking costs and travel + waiting + treatment

times (in hrs) for receiving treatment [17]

^c ^Costs of 1 km = € 0.16, 1 h parking = €2.50 €, 1 h patient's time = €8.30 [17].

^d ^Valued as outpatient mental health services.

^e ^From DFL 77,00 in 1993, converted into Euro, indexed for 2003 (cf. ) and

rounded.

^f ^Valued as domestic help [17].

Direct non-medical costs are costs patients had to make by traveling to health service providers and parking. These costs were valued at €0,16/km and €2,50/hour parking time. We also added the costs of patients' time spent in travel, waiting and treatment at €8,30 [17].

All costs are estimated for the reference year 2003 and are presented in euros.

### Intervention costs

Table 2 represents the cost of the visiting service. Direct medical costs of the intervention included organizing the visiting service, training of volunteers, supervision of the volunteers and the intake by the coordinator of the visiting service (either a paid social worker or a volunteer), the costs of phone calls to both volunteers and participants and overhead costs. In order to estimate these costs we used different sources. First, we calculated the annual costs per participant based on the financial paragraph of the annual report of two participating visiting services. Second, the Manual Visiting Services [13], which was used to set up the visiting services, did also include an estimate of annual costs. This estimate was indexed for 2003 in two ways, by means of a Health Care Index and by means of the General Index as reported by Statistics Netherlands [19]. The costs of the coordinator could differ depending on whether the coordinator is paid or not. A paid social worker is more expensive than a volunteer. Since both options were possible, we used examples of both options in the calculation of the intervention costs. Subsequently, we averaged the four different estimates and added time costs for the volunteers valued at €12,45 per visit (visit plus travel time). Together these direct medical costs added up to €453 annually per recipient of the visiting service. Direct non-medical costs were time costs of the participant, valued at €8,30/visit.

**Table 2:** Calculation of intervention costs

**Source**	**Total Costs**	**Period**	**Participants**	**Annual costs per participant**
Visiting Service 1	12,808	36 months	20	213
Visiting Service 2	54,900	24 months	80	343
Manual method 1^a^	3,403	12 months	10	340
Manual method 2^b^	3,200	12 months	10	320
				
Mean Intervention				304
Time Costs Volunteers	12.45/visit	12 months		149
Direct Medical Costs				453
				
Time Costs Participant	8.30/visit	12 months		100

Total Costs Intervention				553

^a ^From DFL 6,000 in 1997, converted into Euro, indexed for 2003 based on Health Care

Index (cf. ) and rounded.

^b ^From DFL 6,000 in 1997, converted into Euro, indexed for 2003 based on General

Index (cf. ) and rounded.

### Analyses

Statistical analysis was guided by some characteristics of our data.

Primarily, our data were not complete. At the 12 month follow-up assessment 14.4% of the data was missing. All analyses were conducted according to the intention-to-treat principle. Therefore, all missing values were imputed. In order to replace the missing values by plausible estimates, we used the regression imputation procedure as implemented in Stata version 9.1 [20].

Secondly, we had to take into account two confounding variables. Despite random allocation to the research conditions, there were two significant differences between the experimental group and the control group at baseline. Participants in the experimental group were on average more lonely and had a worse quality of life at the start of the study. We adjusted for both confounders (by using residualised QALYs), since they were significant predictors of the QALY end-term,.

**Figure 2:**
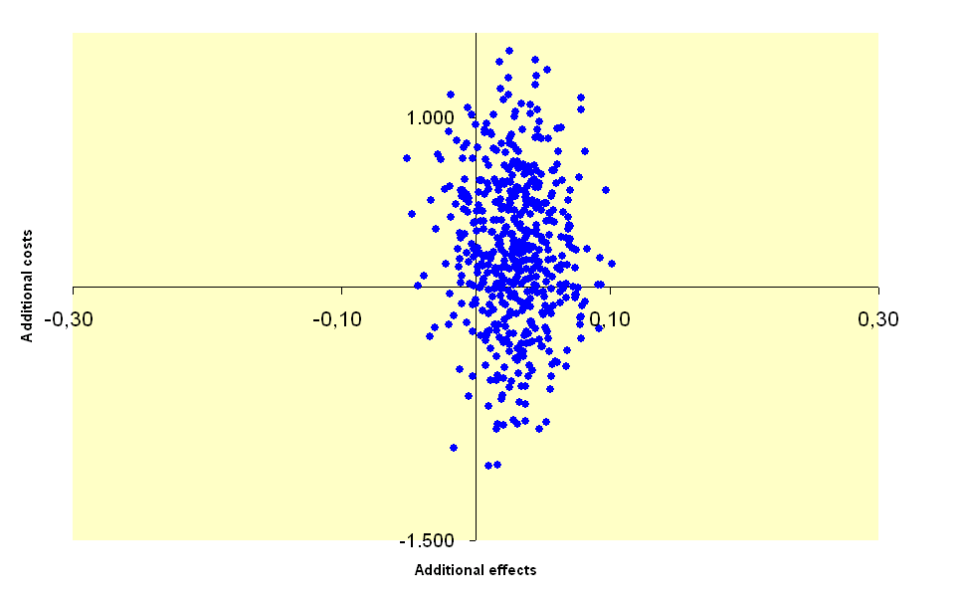
Cost-effectiveness plane: each dot (n = 2,500) represents a bootstrapped cost-utility ratio.

In the cost-utility analysis, we calculated the pre-post changes in costs and the pre-post changes in quality of life in each of the conditions. Then we calculated the incremental cost-utility ratio (ICUR) across the experimental and control conditions, which represents the incremental costs (or savings) per QALY gained in the experimental condition relative to the control condition. Uncertainty was assessed by means of non-parametric bootstrapping (2,500 times) of the data of the individual respondents. The comparison of the simulated ICURs is presented in a cost-utility plane (Figure 2), with differences in costs on the vertical axis and differences in QALYs on the horizontal axis. If the majority of the estimates appear in the top left-hand quadrant of the plane, the intervention results in a loss of quality of life against additional costs as compared with the control condition, which makes the intervention clearly unacceptable from a cost-effectiveness perspective. If the majority of the bootstrapped ICURs appear in lower right-hand quadrant of the plane, the intervention produces more QALYs for less costs than the control condition, which makes the intervention clearly superior from a cost-effectiveness perspective. In the other two quadrants the additional costs or savings have to be weighted against a loss or gain in QALYs.

**Figure 3:**
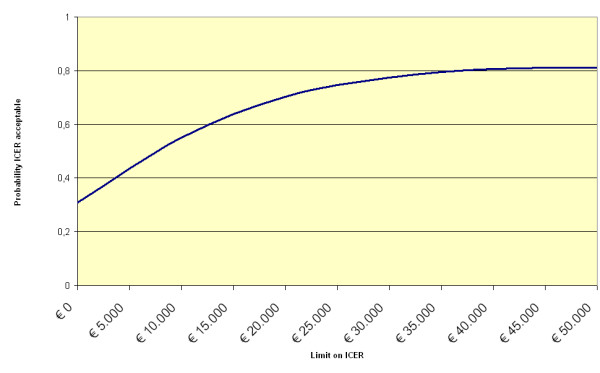
ICUR acceptability curve: probability cost-utility ratio is acceptable given varying thresholds for willingness to pay.

The results of the cost-utility analysis are also presented in a cost-utility acceptability curve (Figure 3). The acceptability curve represents the probability that the intervention is cost-effective, given a varying threshold for the willingness to pay for each QALY gained. Finally, we calculated the Net Monetary Benefit (NMB) for two different ceilings of willingness to pay that are usually applied in the Netherlands (20,000 euro and 80,000 euro). The *net monetary benefit *of a participant is calculated as: *net benefit *= [(willingness to pay) * Δ *effects*] – Δ *costs*.

### Sensitivity analyses

As already mentioned, we conducted our main analyses without the costs attributable to productivity losses since the majority of our sample was not employed. However, since productivity loss is usually the main cost-driver, we repeated all analyses including the costs attributable to productivity losses. Information on the productivity loss was gathered with parts of the Trimbos and institute of Medical Technology Assessment Questionnaire on Costs Associated with Psychiatric Illness (TiC-P) [16]. To evaluate a lost day in a paid job we used age and gender specific friction-costs obtained from Oostenbrink et al. (2004). Friction costs represent the monetary counter-value of production losses that occur during absence from work with a limit to five months [21]. Second, production losses also occur when people are ill, try to work, and are then less efficient. We estimated the number of work cutback days as the number of days actually worked when ill, multiplied by a self-reported inefficiency score, which ranges between 0 and 1 (0 = as efficient as when in good health, 1 = totally inefficient). Again, we used friction costs to valuate these production losses.

## Results

### Sample

The sample consisted of 138 widows (63.8%) and 78 widowers (36.2%). The age of the participants ranged from 50 to 92 years (Mean = 68.8; Sd = 9.3) and the participants had received 13 years of education on average. Duration of widowhood varied from 2 to 14 months (Mean = 7.9; Sd = 1,9). As already mentioned, participants in the visiting service group differed significantly from participants in the control group on two variables: participants in the visiting service group reported more feelings of loneliness than in the CAU group (Mean = 7.1; Sd = 3.0 versus mean = 6.0; Sd = 2.9; t = -2.66; p = 0.008) and a worse health-related quality of life at baseline (EQ-5D utility score mean = 0.76; Sd = 0,25 versus mean = 0.83; Sd = 0.18; t = 2.19; p = 0.030). There was no significant difference in loss to follow-up rates between the research conditions. Furthermore, completers did not differ from non-completers on any of the baseline variables, which indicated that loss to follow-up was at random.

### Quality of Life

Participants in the visiting service group demonstrated a significant improvement in health-related quality of life (EQ-5D utility score at baseline mean = 0.76 (s.d. = 0,25); EQ-5D utility score at 12 months follow-up mean = 0.80 (s.d. = 0.18); Difference mean = 0.04 (s.d = 0.02) QALY gained; t = -2.273; p = 0.025). Participants in the control group did not (baseline mean = 0.83 (s.d. = 0,18); follow-up mean = 0.81 (s.d = 0.21); Difference mean = 0.01 (s.d. = 0.02) QALY lost; t= 0.696; p = 0.488). However, the visiting service group did not significantly differ from the control group in their changes in health-related quality of life over time when we adjusted for both confounding variables (t = 1.29; p = 0.215).

### Costs

Table 3 presents the annual capita costs of both the visiting service group and the control group at baseline and at the 12 months follow-up assessment. In both groups costs increased over time, however these changes in costs were not statistically significant (p = 0.166 in the visiting service group and p = 0.430 in the control group). In the visiting service group, the increased costs included the costs of the intervention (€ 553), but these additional costs were partly compensated for by savings elsewhere in the healthcare and welfare sector. The mean difference of the additional costs was € 210 (s.e. = 363) in favour of the control group, but this difference was not statistically significant (t = -0.579; p = 0.563).

**Table 3:** Annual per capita costs by item and condition

	Annual per capita costs (Direct Medical and Direct Non-Medical) in €					
	Experimental Group (n = 110)			Control Group (n = 106)		

	t0	t2	Diff. t0-t2	t0	t2	Diff. t0-t2

Health service type	Mean (sd)	Mean (sd)	Mean (sd)	Mean (sd)	Mean (sd)	Mean (sd)
						
Medical doctor	255 (328)	245 (342)	-11 (420)	199 (328)	265 (393)	66 (426)
Medical specialist	615 (1534)	490 (1091)	-125 (1710)	308 (835)	437 (1087)	129 (1100)
Regional mental health service	169 (860)	115 (438)	-54 (978)	38 (273)	82 (471)	44 (548)
Regional addiction service	0 (0)	0 (0)	0 (0)	0 (0)	0 (0)	0 (0)
						
Mental Hospital – Outpatient	0 (0)	0 (0)	0 (0)	0 (0)	0 (0)	0 (0)
Mental Hospital – Day care	0 (0)	0 (0)	0 (0)	0 (0)	0 (0)	0 (0)
Mental Hospital – Inpatient	0 (0)	0 (0)	0 (0)	0 (0)	0 (0)	0 (0)
						
General Hospital – Outpatient	10 (105)	27 (151)	17 (186)	0 (1)	16 (110)	16 (110)
General Hospital – Day care	0 (0)	0 (0)	0 (0)	0 (0)	0 (0)	0 (0)
						
Teaching Hospital – Outpatient	0 (0)	0 (0)	0 (0)	0 (0)	0 (0)	0 (0)
Academic Hospital – Day care	0 (0)	0 (0)	0 (0)	0 (0)	0 (0)	0 (0)
						
Private practice psychotherapist	116 (966)	15 (119)	-101 (972)	36 (364)	26 (171)	-9 (405)
Social worker	62 (268)	46 (227)	-16 (334)	54 (291)	42 (186)	-12 (326)
Physiotherapist	298 (810)	322 (931)	24 (1007)	370 (1100)	249 (652)	-121 (952)
Alternative Healer	10 (59)	18 (139)	8 (152)	7 (70)	27 (100)	20 (102)
Self-Help	0 (0)	47 (298)	47 (298)	10 (82)	40 (198)	30 (217)
						
Home care, nursing	1169 (2346)	1261 (2460)	92 (1386)	1068 (2076)	1151 (2078)	83 (1383)
Home care, domestic	55 (491)	35 (246)	-20 (350)	32 (200)	9 (82)	-24 (141)
Informal care (family, friends)	37 (146)	18 (81)	-19 (141)	73 (484)	32 (162)	-42 (512)
						
Antidepressants	14 (60)	13 (68)	-1 (35)	1 (13)	1 (13)	0 (0)
Anxiolytics	7 (23)	6 (18)	-1 (23)	6 (20)	6 (20)	0 (23)
Hypnotics	10 (24)	9 (18)	-1 (20)	4 (14)	5 (14)	1 (13)
						
Total without intervention	2829 (3837)^a^	2666 (3333)^b^	-163 (2938)^d^	2209 (2757)^a^	2389 (2988)^b^	180 (2346)^d^
						
**Intervention: Visiting Service**	**0 (0)**	**553 (0)**	**553 (0)**	**0 (0)**	**0 (0)**	**0 (0)**

Total with intervention	2829 (3837)^a^	3220 (3333)^c^	390 (2938)^e^	2209 (2757)^a^	2389 (2988)^c^	180 (2346)^e^

^a ^No significant difference between total costs at baseline (t0) in the experimental group and total costs at baseline (t0) in

the control group.

^b ^No significant difference between total costs without the costs of the intervention at 1-year follow-up (t2) in the

experimental group and total costs at 1-year follow-up (t2) in the control group.

^c ^Total costs including the costs of the intervention at 1-year follow-up (t2) in the experimental group differ significantly

from the total costs at 1-year follow-up (t2) in the control group at p < 0.10 (p = 0.055).

^d ^No significant difference between the cost difference (t2 - t0) without the costs of the intervention in the experimental

group and the cost difference (t2 - t0) in the control group.

^e ^No significant difference between the cost difference (t2 - t0) including the costs of the intervention in the experimental

group and the cost difference (t2 - t0) in the control group.

### Cost-utility

The incremental cost-utility ratio was calculated as (ΔCosts_E _- ΔCosts_C_)/(ΔQALY_E _- ΔQALY_C_), where ΔCosts represents the average additional per capita costs and ΔQALY represents the number of QALYs gained over time, controlled for both confounding variables, in both the visiting service group (E) and the control group (C). Substitution yields a cost-utility ratio of (390 - 180)/(0.01 - (-0.02)) = 6,827. This means that for each QALY gained by offering the visiting service, the additional costs amount to € 6,827. Bootstrapping of the data of the individual respondents yields a median ICUR of € 4,123 (95% Confidence Interval : – €627,530 – €668,056).

The incremental cost-utility ratio is surrounded by a certain amount of uncertainty, which is presented in the cost-effectiveness plane (Figure 2). Each dot of the cost-effectiveness plane represents a bootstrap replication (n = 2,500) of the incremental cost-utility ratio; 28% of the dots are in the lower right-hand quadrant, indicating a 28% probability that the visiting services generates better health effects against lower costs; there is a 5% probability that the visiting service generates worse outcomes against higher costs and a 1% probability that the visiting service generates worse outcomes against lower costs. However, most dots (59%) are in the upper right-hand quadrant, indicating better outcomes against higher costs.

### Acceptability

The acceptability curve for the incremental cost-utility ratio is presented in Figure 3. The visiting service had a probability of 31% of being more acceptable than the comparator condition from a cost-effectiveness point of view under the conservative scenario that there is no willingness to pay for a gain of one QALY. However, people are generally willing to pay for a QALY gained. When the willingness to pay is raised to € 10,000, the visiting service has a probability of 55% of being cost-effective compared with the informational brochure. Generally, the willingness to pay for a QALY gained by preventive interventions is approximately €20,000, and at this threshold the visiting service has a probability of 70% of being more acceptable than CAU.

### Net Monetary Benefit

Given a willingness to pay of 20,000 Euro for a QALY gained, the Net Monetary Benefit is: NMB = 20,000 * 0.031 – 210 = 410. Given a willingness to pay of 80,000 Euro for a QALY gained the Net Monetary Benefit is: NMB = 80,000 * 0.031 – 210 = 2270.

### Sensitivity analyses

When the indirect costs related to the production losses are included, the incremental cost-utility ratio is €11.239. Bootstrapping of the data yields a median ICUR of € 6,151 (95% Confidence Interval : – €205,706 – €222,067). The distribution of the bootstrapped ICURs over the cost-effectiveness plane is as follows: 63% of the ICURs fall in the upper right-hand quadrant indicating that better effects are obtained against higher costs, 5% fall in the upper left-hand quadrant indicating that the visiting service is inferior, 1% fall in the lower left-hand quadrant indicating that the visiting service has worse clinical outcomes against lower costs, and 24% of the bootstrapped ICURs fall in the lower right-hand quadrant, implying that the visiting service is dominant, because it generates better outcomes against lower costs than the control condition. Under these circumstances, the visiting service has a probability of 27% of being acceptable when the willingness to pay equals zero. When the willingness to pay is increased to € 10,000, and € 20,000, the probability of the visiting service being more acceptable than the control condition increases to 49% and 64% respectively.

## Discussion

We conducted a cost-utility analysis with health-related quality of life as clinical end term. Health related quality of life was measured with the EQ-5D which is a generic measure of health status. As only one of the five components of the EQ-5D has a psychological nature, it is sometimes debated whether the use of this measure is justified in the evaluation of psychological interventions. We believe that it is. First of all, if an individual reports 'extreme problems' on the mental health dimension (anxiety/depression) of the EQ-5D, without any other problems, this health status is still evaluated as 0.36954 by the Dutch general population [15], which represents poor health. By resolving these 'extreme problems' in mental health, effective psychological interventions are able to demonstrate changes in QALYs. Secondly, the use of a generic measure of health-related quality of life enables us to compare the all kinds of interventions on their cost-effectiveness. And although most effective medical procedures usually demonstrate larger improvements in QALYs than psychological interventions, their costs are usually much higher as well. Psychological interventions therefore do not need to result in large changes in QALYs to be cost-effective.

In this study we evaluated the cost-effectiveness of a visiting service for older widows and widowers. The visiting service is a selective preventive intervention. Selective bereavement interventions are directed towards bereaved individuals with a high risk profile. Bereaved individuals with a high risk profile are more likely to experience an abnormal form of grief. The visiting service focussed on loneliness as risk factor. Besides selective bereavement interventions, there are also universal bereavement interventions and indicated bereavement interventions, which are respectively directed towards all bereaved persons and persons who already are experiencing abnormal bereavement. Besides some positive effects of universal prevention for bereaved children, there is hardly any evidence for the effectiveness of universal bereavement interventions [9]. Screening for high risk seemed to increase the efficacy of bereavement interventions. Some studies on selective bereavement interventions demonstrated modest effects, although there were some indications that this is only temporary [9]. Indicated interventions generally seem to lead to favourable results, both for bereaved individuals suffering from complicated grief and bereaved individuals suffering from bereavement-related depression [9]. Given that these bereavement interventions differ in nature and clinical effectiveness, results of this study should not be generalised to indicated interventions or treatment for bereavement related disorders, which are usually administered by a therapist instead of a volunteer and clearly differ from selective interventions like the visiting service.

### Main findings

The experimental group demonstrated a small improvement in health-related quality of life after the intervention. This improvement was absent in the control group. However, since the baseline scores in the control group were significantly higher, there was less possibility for improvement, and when we controlled for this 'false start' the differences in effects on health-related quality of life were no longer significant, which should be read as a warning against overly optimistic interpretations of our data. In both groups, the total costs were higher at the 12 months follow-up assessment than at baseline and the additional costs were somewhat higher in the experimental group than in the control group, although the difference was not significant. Overall, the experimental group demonstrated slightly better results against slightly higher costs. Whether the visiting service is acceptable depends on the willingness to pay: at a willingness to pay equal to zero, the visiting service has a probability of 31% of being acceptable; beyond €20,000, the visiting service has a probability of 70% of being acceptable.

### Limitations

We have to place these findings in the context of the limitations of our study. There are several factors that limit the generalizability of our findings. First, this study is conducted alongside a randomized clinical trial on a visiting service for older widowed individuals with sufficient power to detect changes in clinical outcomes. However, the study was underpowered to detect changes in costs, which usually have large standard errors. Therefore, we took a probabilistic course indicating the likelihood that the intervention was superior from a health economic point of view. The second limitation is the high initial non-response. Only 11.4% of the approached widows and widowers returned the informed consent form. Although part of the non-response is caused by individuals not eligible for the study, both by individuals suffering from full-blown psychiatric disorders and by individuals without feelings of loneliness, it is unlikely that this applies for the complete non-response. Given the absence of information on those widows and widowers not participating in the study, the representativeness of the sample is not clear. One of the risks of studying a vulnerable population is self-selection of the least vulnerable individuals. The results of this study indicate that this probably also applies to a certain extent to our sample. Although the average utility score of our sample was significantly lower at baseline (mean = 0.79; s.d. = 0.22) than the average utility score of the general population (0.88), the average baseline score of our sample did not correspond to high distress either. Furthermore, our data were not complete. At the 12 month follow-up assessment, 14.4% of the data was missing. Although a loss to follow-up of 14% is not much, considering the period of 1 year, the imputation of missing values could still have distorted the results. However, completers did not differ from non-completers on any of the baseline variables, which suggested that loss to follow-up was completely at random. Another limitation was the difference at baseline between the visiting service group and the control group. Despite random allocation to the research conditions, respondents of the visiting service group were more lonely (mean loneliness score was 7.1 (s.d. = 3.0) in the experimental group compared to 6.0 (s.d. = 2.9) in the control condition; t = -2.66 and p = 0.008) and had a worse quality of life (mean 0.76 QALY (s.d. = 0.25) in the experimental group compared to 0.83 QALY (s.d. = 0.18) in the control condition; t = 2.19 and p = 0.030). Although we controlled for both confounding variables in our analyses, our results could have been biased. In addition, instead of monitoring health care utilisation over the entire year of interest in our study, which would clearly imply a large burden on the respondents, we made the simplifying assumption that the health care utilisation during the 4 week period that was assessed with the TIC-P could be interpolated to 1-year estimates from the baseline and follow-up measures. Although we expect that potential bias in these estimates are of similar magnitude in both trial conditions and therefore cancel each other out, the changes in health care costs over time are presumably more complex than is assumed under our model. Because of these limitations, the results of this study should be considered with caution.

## Conclusion

Despite its limitations, this study still offers new information on the potential benefits of bereavement interventions by (trained) volunteers. This study indicates that even in the absence of clinical effectiveness of a bereavement intervention its cost-effectiveness could still be acceptable. However, the acceptability of the visiting service we evaluated, depended mainly on the willingness to pay. Beyond a willingness to pay of €8,000, the visiting service has a probability of 50% of being more cost-effective than the control condition. At lower levels of willingness to pay it is more likely that the visiting service is not cost-effective than that the visiting service is superior.

We assumed that bereavement interventions could be cost-effective because widows and widowers that have been offered targeted support by volunteers will make less use of health care services, the latter being considerably more expensive than the attention of (trained) volunteers. Our data suggests that widowed individuals in the experimental group did indeed make less use of health care services. In the experimental group, total costs without the intervention costs decreased while costs in the control group increased. However, these savings were not large enough to compensate for the intervention costs. In this study, the intervention was still more expensive overall than the control condition.

We already know that bereavement interventions like the visiting service do not produce large benefits in terms of public mental health when targeted towards the entire population of all widowed individuals [[8,9], Onrust et al., submitted]. Based on this cost-utility analysis we can now add that bereavement interventions like the visiting service will also not produce large benefits from the health economic point of view, when targeted towards the entire population of all widowed individuals. Presumably, those widowed individuals that are able to adjust relatively well over time and do not need a specific intervention to regain pre-bereavement levels of functioning, do not make frequent use of the health care services related to their bereavement as well. In light of our findings, we recommend that in depth analyses are conducted to identify who benefits most from this kind of interventions, and in what subgroups the incremental cost-utility is best. In the future bereavement interventions are then best directed to these groups.

## Competing interests

The authors declare that they have no competing interests.

## Authors' contributions

SO was the trial's principal investigator, coordinated data-collection, conducted the analysis and wrote the manuscript. FS supervised the analysis, was the research team's advisor with regard to statistical and economic aspects and assisted with writing the manuscript. GW supervised the trial, coordinated the data-collection and assisted with writing the manuscript. JvdB was the research team's advisor with regard to clinical aspects and supervised the writing of the manuscript. PC designed the study and supervised the writing of the manuscript. All agree with the contents of the manuscript.

## Pre-publication history

The pre-publication history for this paper can be accessed here:


